# Comparative Transcriptome Landscape of Mouse and Human Hearts

**DOI:** 10.3389/fcell.2020.00268

**Published:** 2020-04-22

**Authors:** Tatsuya Anzai, Takanori Yamagata, Hideki Uosaki

**Affiliations:** ^1^Division of Regenerative Medicine, Center for Molecular Medicine, Jichi Medical University, Shimotsuke, Japan; ^2^Department of Pediatrics, Jichi Medical University, Shimotsuke, Japan

**Keywords:** cardiomyocyte maturation, transcriptome, comparative transcriptome, maturation marker, ribosome

## Abstract

Transcriptome landscape of organs from mice and humans offers perspectives on the process of how organs develop and the similarity and diversity in each organ between the species. Among multi-species and multi-organ dataset, which was previously generated, we focused on the mouse and human dataset and performed a reanalysis to provide a more specific perspective on the maturation of human cardiomyocytes. First, we examined how organs diversify their transcriptome during development across and within two species. We unexpectedly identified that ribosomal genes were differentially expressed between mice and humans. Second, we examined the corresponding ages of organs in mice and humans and found that the corresponding developmental ages did not match throughout organs. Mouse hearts at P0–3 and human hearts at 18–19 wpc showed the most proximity in the regard of the transcriptome. Third, we identified a novel set of maturation marker genes that are more consistent between mice and humans. In contrast, conventionally used maturation marker genes only work well with mouse hearts. Finally, we compared human pluripotent stem cell-derived cardiomyocytes (PSC-CMs) in maturation-enhanced conditions to human fetal and adult hearts and revealed that human PSC-CMs only expressed low levels of the potential maturation marker genes. Our findings provide a novel foundation to study cardiomyocyte maturation and highlight the importance of studying human samples rather than relying on a mouse time-series dataset.

## Introduction

Understanding the transcriptome landscape of mouse developing hearts provided a compass to navigate how cardiomyocyte mature *in vivo* and a foundation to determine the maturity of mouse pluripotent stem cell-derived cardiomyocytes (PSC-CMs) cultured *in vitro* (Uosaki et al., 2015). Single-cell RNA-sequencing added the details on cell-type specific, spatiotemporal developmental programs in mice hearts, although it lacked fully-matured adult cardiomyocytes in the analysis (DeLaughter et al., 2016). The maturation of cardiomyocytes is a continuous process from early embryo to adult, but the maturation of PSC-CMs arrested at the late-embryonic stage (Uosaki et al., 2015), which demanded more information on cardiomyocyte maturation during postnatal maturation. Due to the limited availability of human heart samples at late-embryos to early ages, only transcriptome datasets of fetal and adult human hearts were evaluated in the past (Kuppusamy et al., 2015; Uosaki and Taguchi, 2016). Although many genes shared distinct expression patterns from fetus to adult in mouse and human hearts (Uosaki and Taguchi, 2016), the expression kinetics of maturation-related genes during the entire maturation process in human hearts were largely unknown.

Recently, time series of the transcriptome for six mammals and a bird, with seven organs, were conducted and provided from the other group (Cardoso-Moreira et al., 2019). While the dataset still lacks late-embryonic human samples, it offers the most complete dataset of human organs from early embryos to adults. Here, we hypothesized that detailed analyses of the dataset could reveal not only how the transcriptome of human hearts progresses but also the expression kinetics of maturation-related genes. In this study, we reanalyzed the dataset to obtain more accurate information about the transcriptome landscape of mice and humans, especially in hearts from embryonic to adult stage and determined expression kinetics of maturation-related genes in human hearts.

## Materials and Methods

### Data Sets

The datasets analyzed for this study can be found in E-MTAB-6798 (mouse)^1^ and E-MTAB-6814 (human)^2^. We used normalized data with reads per kilobase per million mapped reads (RPKM) in the repository. For the analysis of gene expressions in PSC-CMs, we obtained read counts data from GSE62913^3^ and normalized to transcripts per million reads (TPM).

### Bioinformatics Analysis

We performed most of the analyses using the statistical software R version 3.6.1 (R Core Team, 2016). To identify orthologs, we used biomaRt and homologene (Durinck et al., 2009; Mancarci, 2018). For the data analysis, we first performed principal component analysis (PCA) with logarithmically converted data [log(RPKM + 1)] using prcomp function in R. To generate graphs and plots, we used ggplot2 (Wickham, 2016). For the 3D plots, we used plotly for R (Sievert, 2020) and exported as hypertext markup language (HTML, Supplementary Data S1–S9). To define developmental stages, we used k-means clustering and designated samples in each organ to five clusters unless noted otherwise. To identify differentially expressed genes (DEGs) in multigroup comparison settings, we used edgeR and performed generalized linear model and likelihood ratio test (GLM-LRT) (Robinson et al., 2010; McCarthy et al., 2012). Genes with false discovery rate (FDR) < 0.01, *P*-value < 0.01, log2 fold changes > 1, and log2 averaged RPKM > 3 were considered as DEGs. DEGs were further clustered using c-means fuzzy clustering with Mfuzz (Futschik and Carlisle, 2005; Kumar and Futschik, 2007). Genes with membership score > 0.5 are considered as members of a cluster. For gene ontology (GO) analysis, we used clusterProfiler and DOSE (Yu et al., 2012, 2015). We used cut-offs of 0.1 for both *P*-value and *Q*-value, and the FDR (also known as BH) adjustment was applied to adjust *P*-value. Top five GO terms with the lowest *P*-value were reported though some of them were not significantly enriched. To draw a gene-concept network, cnetplot function in enrichplot package was used. To define a candidate marker gene for maturation from the gene clusters 1 and 4 (upregulated in adults compared to embryos), we set criteria of RPKM > 100 in both mouse and human adult heart with > 10-fold changes when compared the adult to early embryonic hearts.

## Results

### Global Transcriptome Analysis Comparing Organ Scale Maturation

**FIGURE 1:**
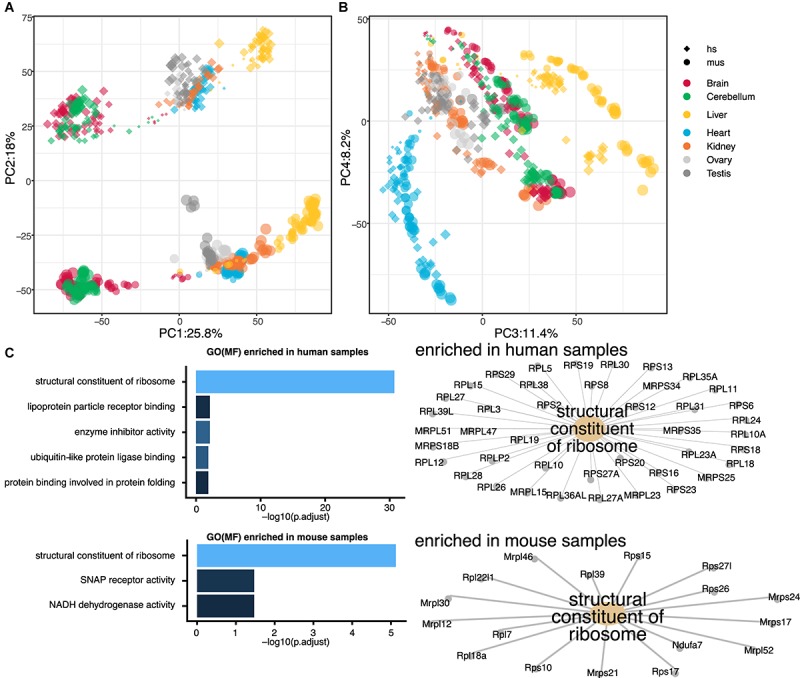
Global transcriptome analysis of seven organs from mouse and human. **(A**,**B)** Principal component analysis (PCA) of seven organs from mouse and human. Sizes of dots indicate ages of samples [small dot, young; big dot, old. Ages ranging 4 weeks post conception (wpc)–senior of human and embryonic day (E) 9.5–postnatal (P) 63 of mouse]. Shapes of dots indicate species [diamond, human (hs); circle, mouse (mus)]. Color of each point indicates organs (red, brain; green, cerebellum; yellow, liver; blue, heart; orange, kidney; light gray, ovary; dark gray, testis). Principal component (PC) 1, 3, and 4 distinguish the organs while PC2 separate mouse and human. **(C)** Gene ontology (GO) analysis of PC2 axis. Top five GO terms for molecular function (MF) enriched in human and mouse organs are shown as bar graphs (left). In both mouse and human, GO term “structural constituent of ribosome” was highly enriched, though the enriched genes were different as shown in gene-concept networks. SNAP, synaptosomal-associated protein; NADH, nicotinamide adenine dinucleotide.

Gene expression time series for six mammals and a bird, with seven organs, were performed in the previous study by the other group (Cardoso-Moreira et al., 2019). In this study, we aimed to perform a reanalysis of the dataset to obtain a more focused perspective on the transcriptome landscape, especially in hearts. As the most basic biomedical researches are conducted with mouse models, we decided to focus only on mouse and human rather than all seven species, which would allow us to translate the knowledge with mice into human researches. First, we examined the comparative transcriptome between humans and mice throughout the seven organs. To directly compare the human and mouse dataset, we curated 16,831 genes with 1:1 ortholog in mouse and human using biomaRt and homologene. Then, we performed PCA using all of the orthologs. While PC1 separated the samples based on the germ layer as previously shown, PC2 differentiated the samples by species (Figure 1A and Supplementary Data S1). This notion was unexpected as we expected organ-to-organ variations are more distinctive than species differences. With the combination of PC1, 3, and 4, the samples were distinguished by organs and developmental stages (Figure 1B and Supplementary Data S2). Clearer separations were observed with combinations of PC1 to PC3 in the PCA plots within single species (Supplementary Data S3, S4). To ask what makes human and mouse different in the transcriptome landscape, we performed GO analysis. Interestingly, GO term “structural constituent of ribosome” was highly enriched in both positively (human-specific) and negatively (mouse-specific) weighted genes responsible for PC2-axis (Figure 1C), however, the member genes enriched in the GO term for each species were distinctive. This result implicates ribosomal function might distinguish mice and humans regardless of organs.

### Developmental Stage Correspondences of Each Organ

**FIGURE 2:**
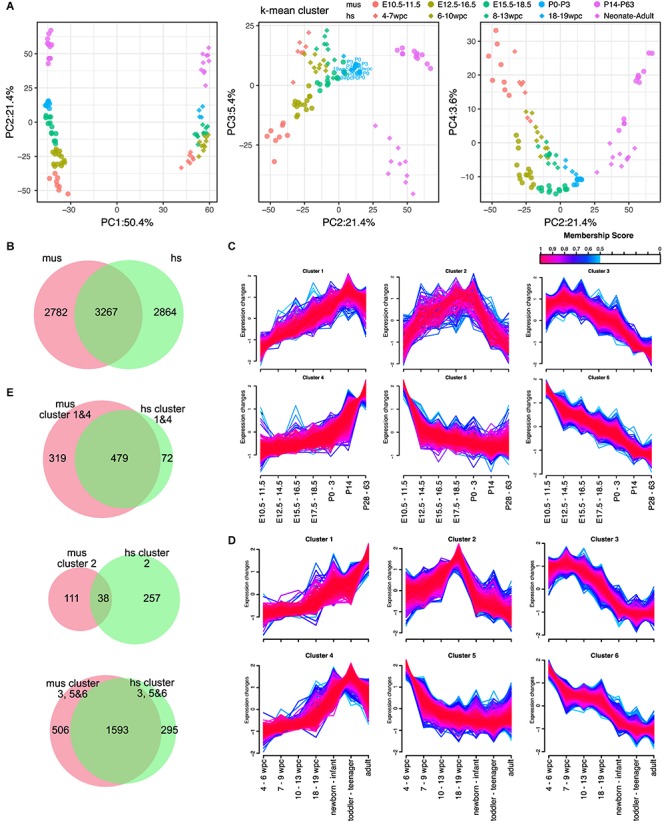
Expression dynamics of DEGs during the maturation in hearts. **(A)** PCA of hearts from mouse and human. Shapes of dots represent species [diamond, human (hs); circle, mouse (mus)]. Color represents corresponding ages based on k-mean clusters as shown in figure. Ages shown in PCA plots indicate the ages of each point that is the closest dots between mouse and human. **(B)** Venn diagram of differentially expressed genes (DEGs) in mouse (mus) and human (hs). **(C,D)** Fuzzy clustering of DEGs in mouse **(C)** and human **(D)** with membership score of at least 0.5. Color indicates membership score of each gene in a cluster. **(E)** Venn diagrams of member genes in mouse and human. Gene clusters upregulated in adult (cluster 1 and 4), temporally upregulated (cluster 2), and downregulated in adult (cluster 3, 5, and 6) are shown.

Next, we aimed to define the corresponding developmental stages of each organ between mice and humans. For embryonic development, the Carnegie stages are often used (de Bakker et al., 2016). However, it is unknown if each organ developed the same manner in a body between humans and mice. To unveil this, we performed the PCAs of individual organs. As we expect, PC1 and PC2 are major explanatory variables of species and developmental stages, respectively, in most of the organs except testes (Figure 2A, Supplementary Figure S1, and Supplementary Data S5–S9). Proximity in a PCA plot indicates transcriptional similarity among samples. With *k-mean* clustering, we classified each organ of mouse and human to five clusters. Although the phylotypic stage in the developmental hourglass model is considered at the mid-embryonic stage (Irie and Kuratani, 2011), PCA plots of individual organs displayed more proximity at some point around birth. Interestingly, the time of the most proximate varies organ-to-organ. For example, human hearts at 19 weeks post conception (wpc) were closest to mouse hearts at postnatal day (P) 0–3 (Figure 2A and Supplementary Data S5). In contrast, human kidneys at 10–20 wpc and livers at 19–20 wpc were closest to mouse counterparts at P3–P14 and E17.5, respectively (Supplementary Figures S1A,B and Supplementary Data S6, S7). When brains and cerebellums from mouse and human were analyzed altogether, they had similar trajectory up to the late embryonic-neonatal stage and differentiated to more specific to brain or cerebellum later (Supplementary Figure S1C and Supplementary Data S8). Moreover, both mouse and human testes displayed sudden transcriptional changes at the adolescent stages (Supplementary Figure S1D and Supplementary Data S9). These results indicate that every organ has a different developmental speed.

### Differentially Expressed Genes in Heart

The heart is a major target organ for us. We previously demonstrated that the developmental stages of mouse PSC-CMs can be accurately predicted using the microarray-based dataset (Uosaki et al., 2015). However, due to the lack of compatibility across platforms of microarray and limited dataset of human hearts, it is unable to use the method to human PSC-CMs. We also demonstrated that mouse and human transcriptomes change similarly from embryo to adult (Uosaki and Taguchi, 2016), while no time-course study was performed at that point. Here, we examined the transcriptome data of mouse and human hearts to show the trajectory of transcriptome changes in mouse and human hearts. As we demonstrated that there is a specific time-point that an organ from mouse and human becomes more similar than the other time-points. Thus, we identified DEGs among three clusters – the earliest developmental clusters (E10.5–11.5 of the mouse hearts; 4–7 wpc of the humans), the closest clusters in between (P0–3 for mouse hearts; 18–19 wpc of humans), and the most aged clusters (P14–63 of mouse hearts; neonate–old mid age of humans). We identified more than 6,000 genes are differentially expressed at least one comparison among the clusters in both mouse and human hearts (Figure 2B and Supplementary Figures S2A–C). Among them, 3,267 genes were overlapped in mouse and human DEGs of hearts (Figure 2B). To test if expression trajectory is similar during heart development in mouse and human, we clustered the common DEGs of hearts using c-means fuzzy clustering. We identified six DEG clusters in mouse hearts (Figure 2C), which included steadily increased to adult (cluster 1), adult heart-specific (cluster 4), steadily decreased (clusters 3 and 6), early embryonic heart-specific (cluster 5), and transient upregulation (cluster 2). We also identified 6 DEG clusters in human hearts (Figure 2D), however, we found gene cluster upregulated in the postnatal-adult hearts (cluster 4) instead of adult heart-specific. Next, we asked if identified clusters shares genes. To test this, we compared genes in clusters 1 and 4 (upregulated in adults compared to embryos), cluster 2 (transiently upregulated), and clusters 3, 5, and 6 (downregulated in adults). Upregulated or downregulated genes were largely shared in mouse and human (Figure 2E), while genes in cluster 2 were almost distinct. To determine gene functions of upregulated or downregulated genes, we performed GO analysis on commonly upregulated or downregulated genes in adult mouse and human hearts. GO analysis revealed that genes enriched in adult hearts were related to mitochondrial activities, such as electron transfer and Nicotinamide adenine dinucleotide (NADH) dehydrogenase activity (Supplementary Figure S3A). In contrast, cell division-related genes were highly enriched in downregulated genes (Supplementary Figure S3A), which is consistent with our previous study (Uosaki et al., 2015). Next, we examined cluster 2s to determine what define the difference in transiently upregulated genes. In mouse hearts (Supplementary Figure S3B), extracellular matrix related GO terms are enriched in the genes specifically expressed in mouse cluster 2. In contrast, no GO term for molecular function (MF) were significantly enriched in human (Supplementary Figure S3C). GO terms for biological process (BP) enriched in the genes of human cluster 2 were related cardiac function (e.g., cardiac muscle cell action potential).

### Marker Genes for Cardiomyocyte Maturation

**FIGURE 3:**
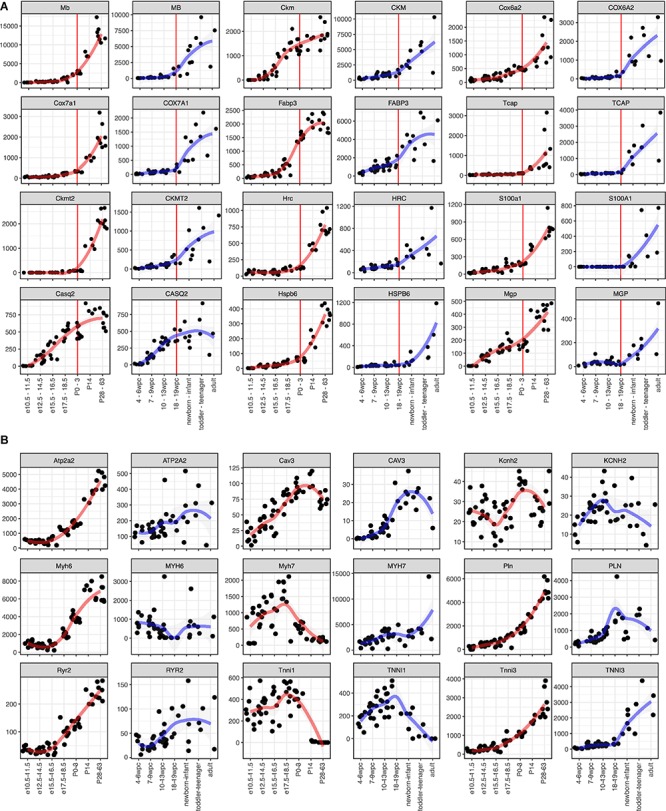
Expression kinetics of the potential maturation marker genes. **(A**,**B)** Time course gene expression profiles. Mouse (left side) and human (right side) data is shown side-by-side. A red (for mouse) or blue (for human) line indicates local regression curve using locally estimated scatterplot smoothing (LOESS). **(A)** Candidate marker genes identified in this study. Red vertical lines indicate P0-3 in mouse and 18–19 wpc in human, which are considered as the same developmental stage. **(B)** Marker genes used in other literatures.

To apply this analysis to human PSC-CMs or cardiomyocyte maturation, identifying maturation markers would help. As most of such markers were identified or used based on the knowledge of mouse development, little is known for usefulness in human studies. Thus, we aimed to identify candidate genes for maturation markers of cardiomyocytes from the commonly upregulated DEGs. The medians of the common genes (479 genes, Figure 2E) in clusters 1 and 4 were 34.0 and 36.6 RPKM at the adult stage in mouse and human, respectively (Supplementary Figure S4A). To select highly expressed genes in adult hearts, we used 100 RPKM in both mouse and adult hearts as the minimum expressions. Next, we examined fold changes between the developmental stages (Supplementary Figures S4B–H) or between the species (Supplementary Figure S4I). Most of the genes showed less than 10-fold changes in any comparison between the stages. 10-fold changes between adult and early embryonic hearts in one species yielded 100-fold changes in the other species (Supplementary Figure S4I), and the genes with 10-fold upregulation in adult from early embryonic hearts include most of the genes that were upregulated in a later stage compared to earlier stages (Supplementary Figures S4B–H). Overall, 43 genes were selected as potential maturation marker genes, which showed fold changes of at least 10 times from the early embryos to adults and higher expression levels (> 100 RPKM) in both mice and human adult hearts (Supplementary Figures S5, S6 and Figure 3A). Considering the expression levels and the consistency throughout the samples and species, we narrow down to 12 genes (Figure 3A). *MB*, *CKM*, *COX6A2, COX7A1, FABP3, and TCAP* were promising candidates for maturation markers as they reached more than 1,000 RPKM in adults, which would be high enough for making a fluorescent marker as well. TNNI3, a conventionally used marker, also displayed the same profile (Figure 3B). We examined nine conventionally used markers for cardiomyocyte maturation, including isoform switches (*MYH7* to 6, *TNNI1* to 3) to ask if these markers are reliable for human (van den Berg et al., 2015; Karakikes et al., 2015; Denning et al., 2016; Ahmed et al., 2020). All but *Kcnh2* displayed expected gene expression profiles in mouse hearts. In contrast, only *CAV3* and *TNNI3* showed consistent profiles as maturation markers for human cardiomyocytes. The expression kinetics of candidate genes varies in mouse and human: some genes (e.g., *MB* and *RPL3L*) started to express earlier in human hearts than mouse hearts at the corresponding stage but others were rather expressed later (e.g., *CKM* and *MGP*). These findings support that the novel set of maturation marker genes can be more reliable than the current set of marker genes.

### Expression of Marker Genes in PSC-CMs

**FIGURE 4:**
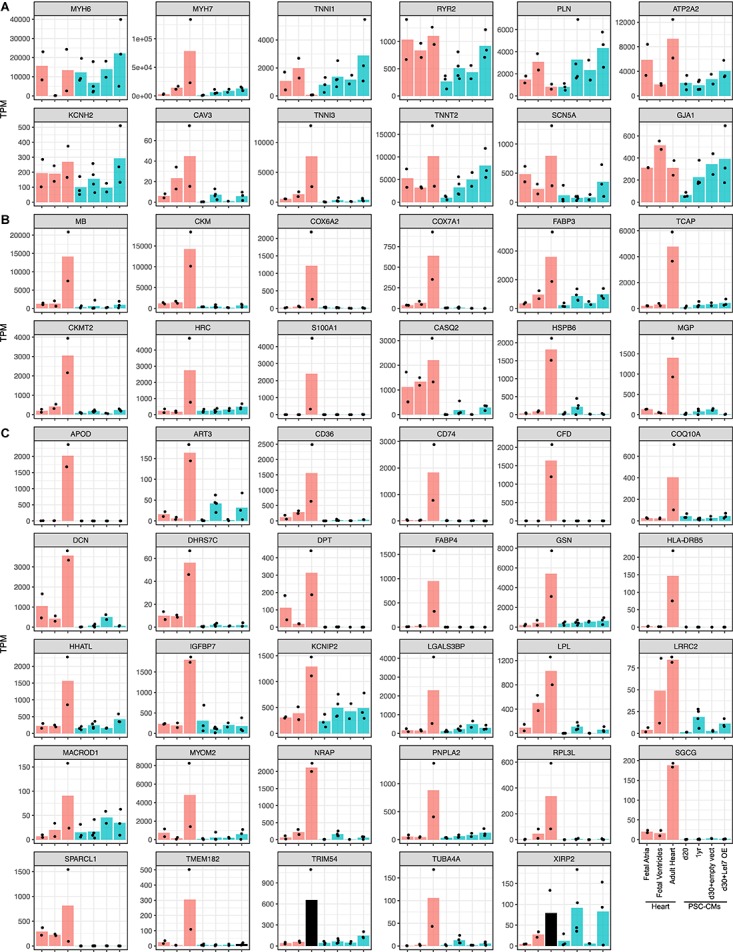
Expressions of the maturation marker genes in PSC-CMs. Graphs of the candidate maturation marker gene expressions in human fetal and adult hearts and PSC-CMs. PSC-CMs at day 20 and day 30 with empty vectors are considered as immature while those at 1 year and d30 with Let7 overexpression vector (OE) are considered as more matured. **(A)** Common maturation marker genes including the isoform switch and the genes used in the original study. **(B)** Top 12 and **(C)** the rest of the candidate maturation marker genes identified in this study. Bars are shown in the mean of transcripts per million reads (TPM) and each dot indicates TPM of each sample.

To ask if the candidate maturation markers could dictate maturation of PSC-CMs, we reanalyzed an RNA-seq dataset include human fetal and adult hearts and PSC-CMs with maturation-enhanced conditions (Kuppusamy et al., 2015). In the previous study, PSC-CMs were cultured for 1 year or treated with Let-7 overexpression vector to enhance the maturation. Among 9 conventional markers or the genes the authors used (Figure 4A), *MYH7*, *ATP2A2*, *CAV3*, *TNNI3*, *TNNT2*, and *SCN5A* were upregulated from fetal ventricles to adult hearts, and *TNNI1* was downregulated. In PSC-CMs, *CAV3*, *TNNT2*, and *SCN5A* along with *KCNH2*, *RYR2*, and *PLN* were upregulated in maturation enhanced condition compared to control, although the expression of *CAV3* and *TNNI3* were relatively low and comparable to the fetal hearts. Low expression of *MYH7* and increased expression of *TNNI1* might indicate PSC-CMs in the maturation-enhanced condition remained in the fetal stages. Next, we examined the expression of the potential novel set of maturation markers (Figures 4B,C). *PERM1* was not found in the dataset, which leaves 42 genes including *TNNI3*. Among 41 genes, *XIRP2* was the only genes expressed at the level of adult hearts in PSC-CMs with the maturation-enhanced conditions. The rest was the level of fetal hearts or lower than that, even after upregulation. The genes expressed from earlier stages of human hearts (e.g., *CASQ2*, *LPL*, and *LRRC2*) displayed relatively higher expression in fetal ventricles and in PSC-CMs with the maturation-enhanced conditions. Taken together, the maturation-enhanced conditions could indeed increase maturation-related genes, but the overall maturation status remained in the fetal stages.

## Discussion

### Identification of Maturation Markers

Cardiomyocyte maturation arrests at an immature state *in vitro*, and discovering a new method to generate mature PSC-CMs is a huge point of emphasis for the last decade (Uosaki et al., 2015; Cho et al., 2017; Ronaldson-Bouchard et al., 2018) after efficient differentiation methods were developed (Uosaki et al., 2011; Tohyama et al., 2013; Burridge et al., 2014). Since cardiomyocyte maturation is generally examined with physiological properties, and/or structural and morphological features, studying mechanisms of maturation is inefficient and little is understood. Moreover, some studies compared gene expression of PSC-CMs and adult human cardiomyocytes with a selected set of genes (Yang et al., 2014a; Karakikes et al., 2015), however, the selection of such genes is based on mouse expression profiles and the expression kinetics of the maturation marker genes during human heart development were unknown. In this study, we discovered a potential novel set of marker genes for maturation, which have similar kinetics in both humans and mice (Figure 3A). In addition, the commonly used marker genes displayed expected profiles in mice, however, they did not have the same dynamics or clearness in humans (Figure 3B). Recently, we developed a fluorescent reporter line for cardiomyocyte maturation (Chanthra et al., 2020). We inserted red fluorescent protein (RFP) into 3’ end of *Myom2* locus to fuse RFP with Myom2 and demonstrated that RFP positive PSC-CMs were more mature than RFP negative cells. We also demonstrated that the isoform-specific effects of laminin on cardiomyocyte maturation. Physiological and morphological analysis revealed Myom2-RFP PSC-CMs resembled postnatal cardiomyocytes, suggesting that 100 RPKM might be enough to detect fluorescence from the endogenous locus, though it must be validated individually. Generating reliable fluorescent reporter lines based on the novel maturation markers would further facilitate researches on cardiomyocyte maturation, and the maturation marker genes we identified in this study can be a candidate to generate them.

### Implication to Disease Modeling With Human PSC-CMs

As we demonstrated, the expressions of the potential maturation-marker genes were remained low in PSC-CMs even after 1-year cultures. Successful cardiac disease models with immature PSC-CMs would be limited to some diseases that display phenotypes from the very early stages of life as the immature PSC-CMs have distinct electrophysiological properties including calcium handling and metabolisms from adult cardiomyocytes (Yang et al., 2014a; Ahmed et al., 2020). Thus, challenges are still generating more mature PSC-CMs to recapture the disease phenotypes of late-onset cardiac diseases. There have been many attempts to achieve this goal with prolonged culture, electrical and mechanical stimulation, hormones and extracellular matrices (Kamakura et al., 2013; Lundy et al., 2013; Nunes et al., 2013; Yang et al., 2014b; Ronaldson-Bouchard et al., 2018; Chanthra et al., 2020), and some of them seemed more successful. However, it is difficult to compare one to the others because there is no definitive evaluation method of cardiomyocyte maturation. With our novel set of potential marker genes, we might be able to develop more accurate methods to define the maturity of PSC-CMs; the methods can be a fluorescent reporter or targeted RNA-seq of the marker genes.

### The Expression Kinetics of Maturation Markers

The overall transcriptome trajectory highlighted that mouse and human hearts are more similar at P0–3 and 18–19 wpc, respectively. Upregulated genes in the course are largely identical and involve in mitochondrial function. These findings are particularly interesting because mitochondria activity turns glycolysis to fatty acid β-oxidation postnatally (Lopaschuk et al., 1992). Although the overall transcriptome of P0–3 mouse hearts is more similar to that of 18–19 wpc human hearts (Figure 2A), expression kinetics of individual maturation marker genes are different. For instance, *Ckm* and *Nrap* start to express in embryonic mouse hearts at E15.5–16.5 and E12.5–14.5, respectively, while their expression becomes evident at 18–19 wpc in human hearts. Moreover, *Ckmt2*, a mitochondrial gene, expressed only after P14 in mouse hearts, but its expression starts mid-gestation in human hearts. These findings suggest that mitochondria might become more active in human fetal hearts.

### Ribosomes Might Define Mouse and Human Differences

The size of somite is determined by the periodic expressions of genes, which is called “segmentation clock.” The oscillation periods are species-specific and regulated not only by sequence differences in the involved genes but also by species-specific cell-autonomous differences in biochemical reaction parameters (Chu et al., 2019; Matsuda et al., 2019; Diaz-Cuadros et al., 2020). We unexpectedly revealed that the members of ribosomal genes are differentially expressed in mice and humans. Since ribosomes translate mRNA to proteins, the expression differences in ribosome genes can be a major determinant of the differences in the speed of the segmentation clock. It requires further researches to fully understand how biochemical reaction parameters are different in mice and humans, including protein synthesis rates (Li et al., 2014).

## Data Availability Statement

The datasets analyzed in this study can be found in the E-MTAB-6798, E-MTAB-6814, GSE62913.

## Author Contributions

TA and HU conducted bioinformatics analyses and wrote the manuscript. TY provided necessary supervision.

## Conflict of Interest

The authors declare that the research was conducted in the absence of any commercial or financial relationships that could be construed as a potential conflict of interest.

## Footnotes

1 https://www.ebi.ac.uk/arrayexpress/experiments/E-MTAB-6798/

2 https://www.ebi.ac.uk/arrayexpress/experiments/E-MTAB-6814/

3 https://www.ncbi.nlm.nih.gov/geo/query/acc.cgi?acc=GSE62913
