# Supplementary material for: Comparative Transcriptome Landscape of Mouse and Human Hearts
Source: Front Cell Dev Biol. 2020 Apr 22;8:268. doi: 10.3389/fcell.2020.00268 (PMC7188931; doi:10.3389/fcell.2020.00268)
Supplement: Supplementary file 2 [file Image_1.PDF]

Supplementary Material

1 Supplementary Data

1.1 Supplementary Data 1

Three dimensional PCA plot (PC1-3) of all tissues from mouse and human.

1.2 Supplementary Data 2

Three dimensional PCA plot (PC1-4, except PC2) of all tissues from mouse and human.

1.3 Supplementary Data 3

Three dimensional PCA plot (PC1-3) of all tissues from mouse.

1.4 Supplementary Data 4

Three dimensional PCA plot (PC1-3) of all tissues from human.

1.5 Supplementary Data 5

Three dimensional PCA plot (PC2-4) of hearts from mouse and human.

1.6 Supplementary Data 6

Three dimensional PCA plot (PC2-4) of kidneys from mouse and human.

1.7 Supplementary Data 7

Three dimensional PCA plot (PC2-4) of livers from mouse and human.

1.8 Supplementary Data 8

Three dimensional PCA plot (PC2-4) of brains and cerebellums from mouse and human.

1.9 Supplementary Data 9

Three dimensional PCA plot (PC2-4) of testes from mouse and human.

2 Supplementary Figures

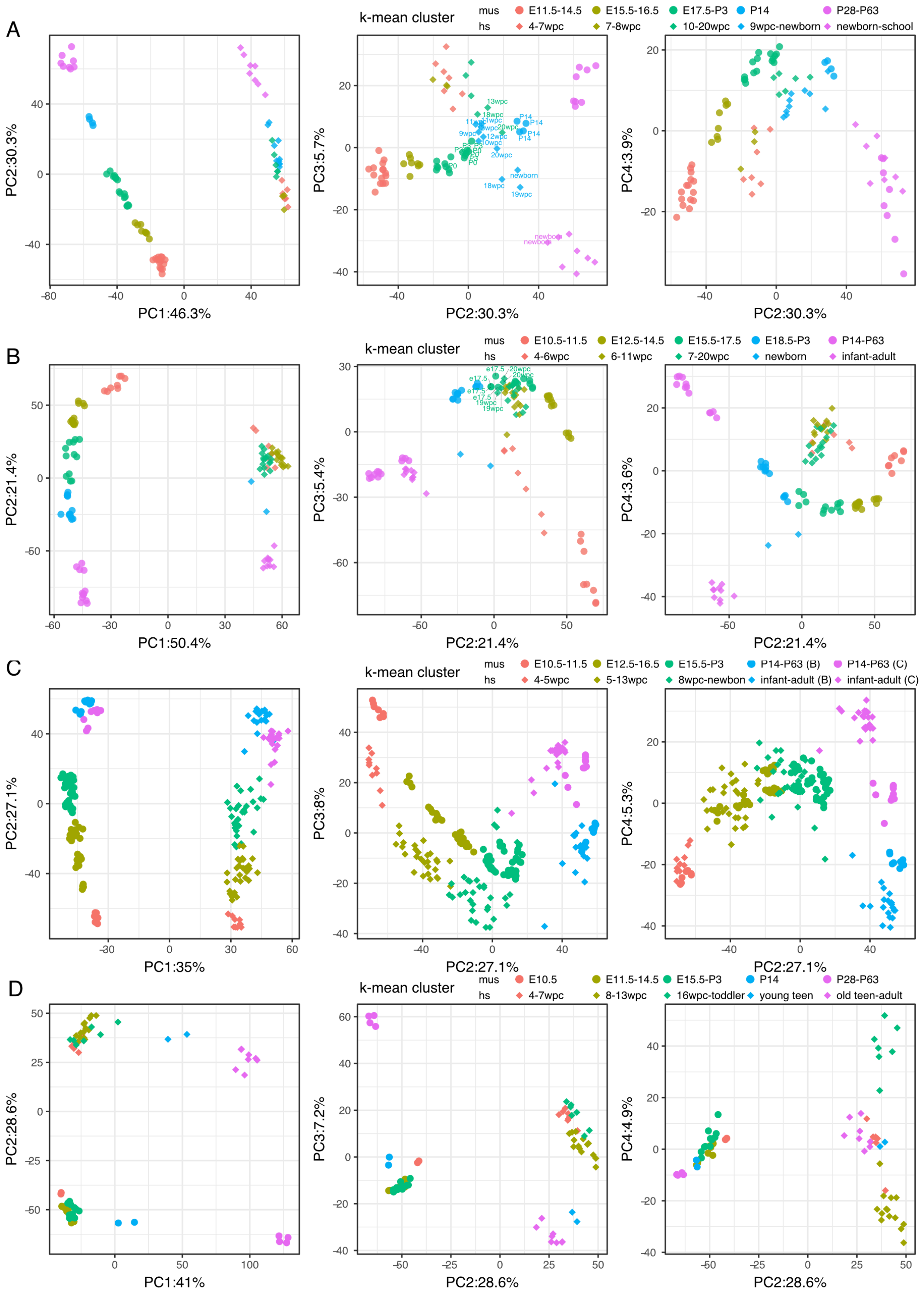

Supplementary Figure 1: Global transcriptome analysis of brains and cerebellums and testes.

PCA plots of kidneys (A), livers (B), brains and cerebellums (C) and testes (D). Shapes and color represent species corresponding ages based on k-mean clusters as shown in the figure. The most aged clusters were separately shown for brains and cerebellums.

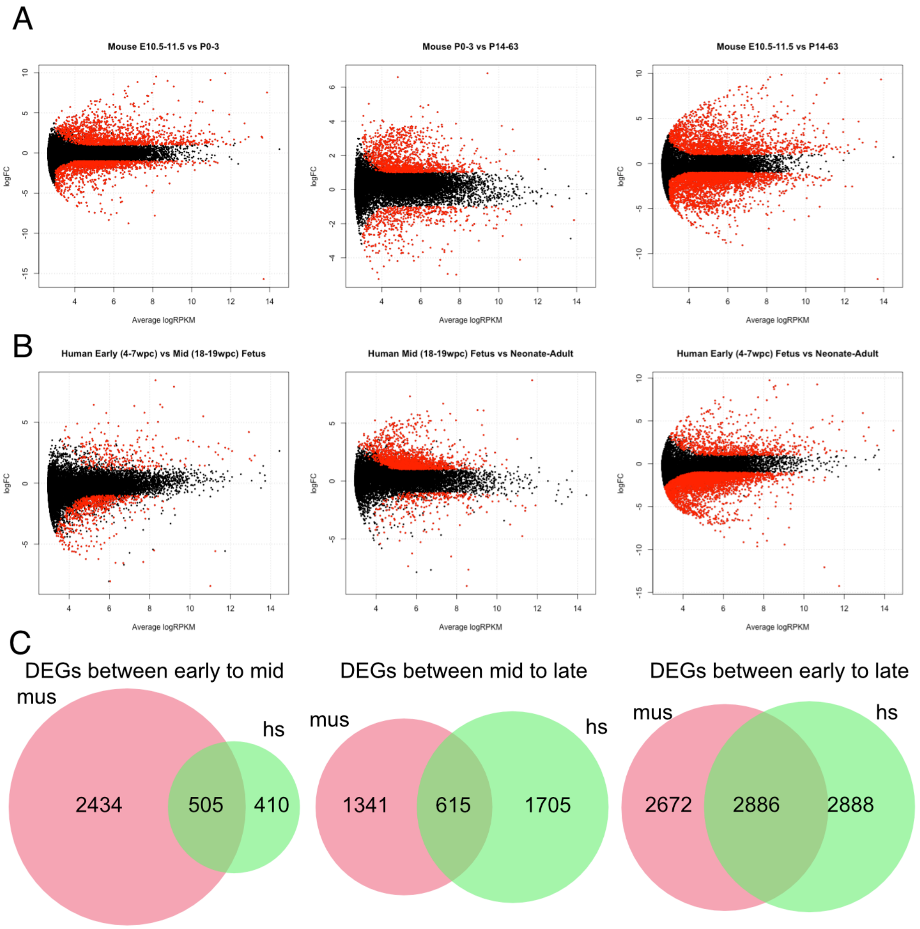

Supplementary Figure 2: Differentially expressed genes in hearts.

(A-B) Smear plot of differentially expressed genes (DEGs) in mouse (A) and human (B) hearts. (C) Venn diagrams of DEGs between early (E10.5-11.5, 4-7 wpc) to mid (P0-3, 18-19 wpc), mid to late (P14-P63, neonate-adult), or early to late in mouse and human hearts.

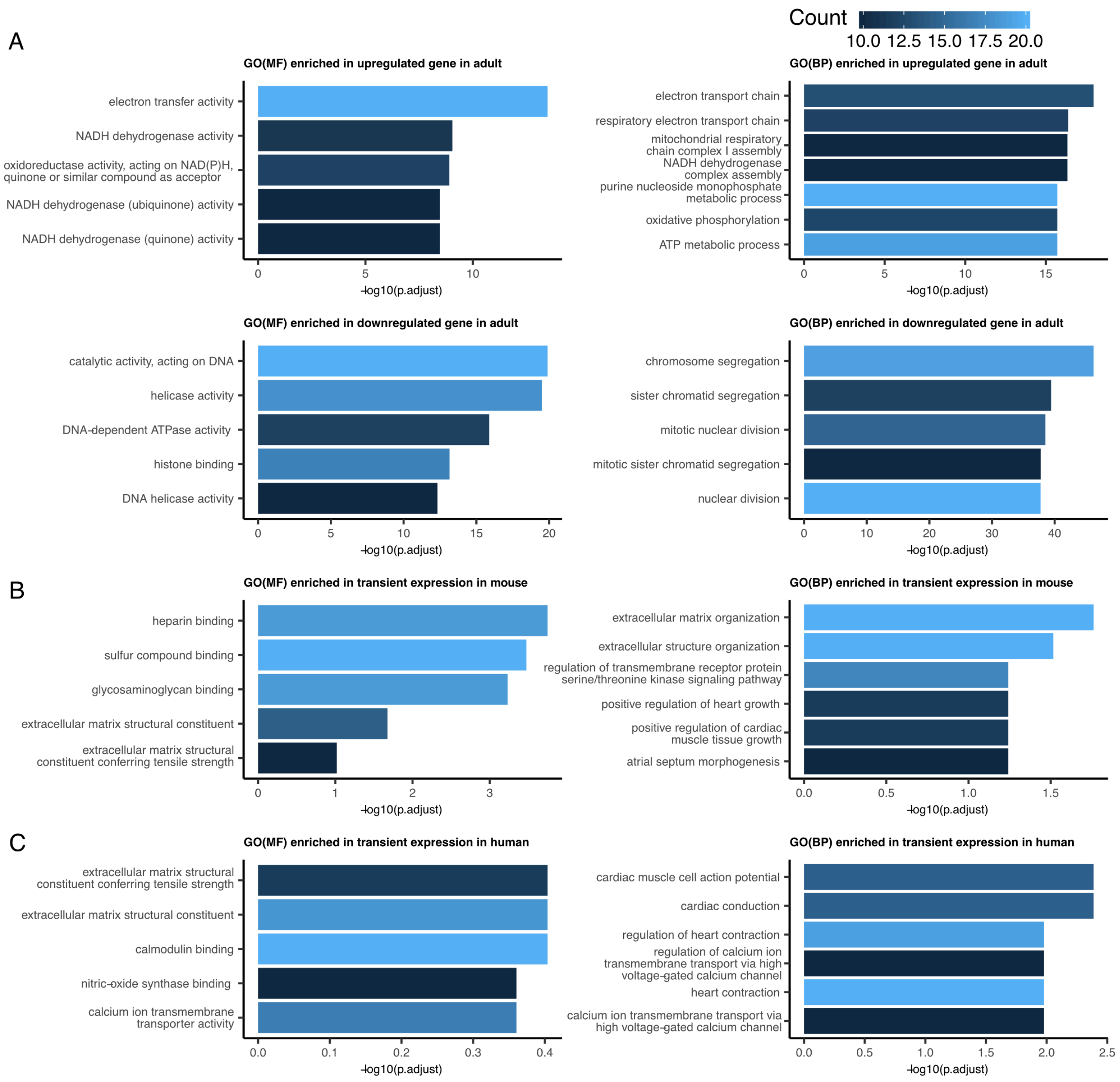

Supplementary Figure 3: GO analysis of differentially expressed genes.

Top 5 GO terms enriched in (A) commonly upregulated or downregulated genes in adult hearts, and transiently expressed genes specifically found in (B) mouse and (C) human. Bar color indicates numbers of genes in the GO terms enriched. Abbreviation: NADH, Nicotinamide adenine dinucleotide.

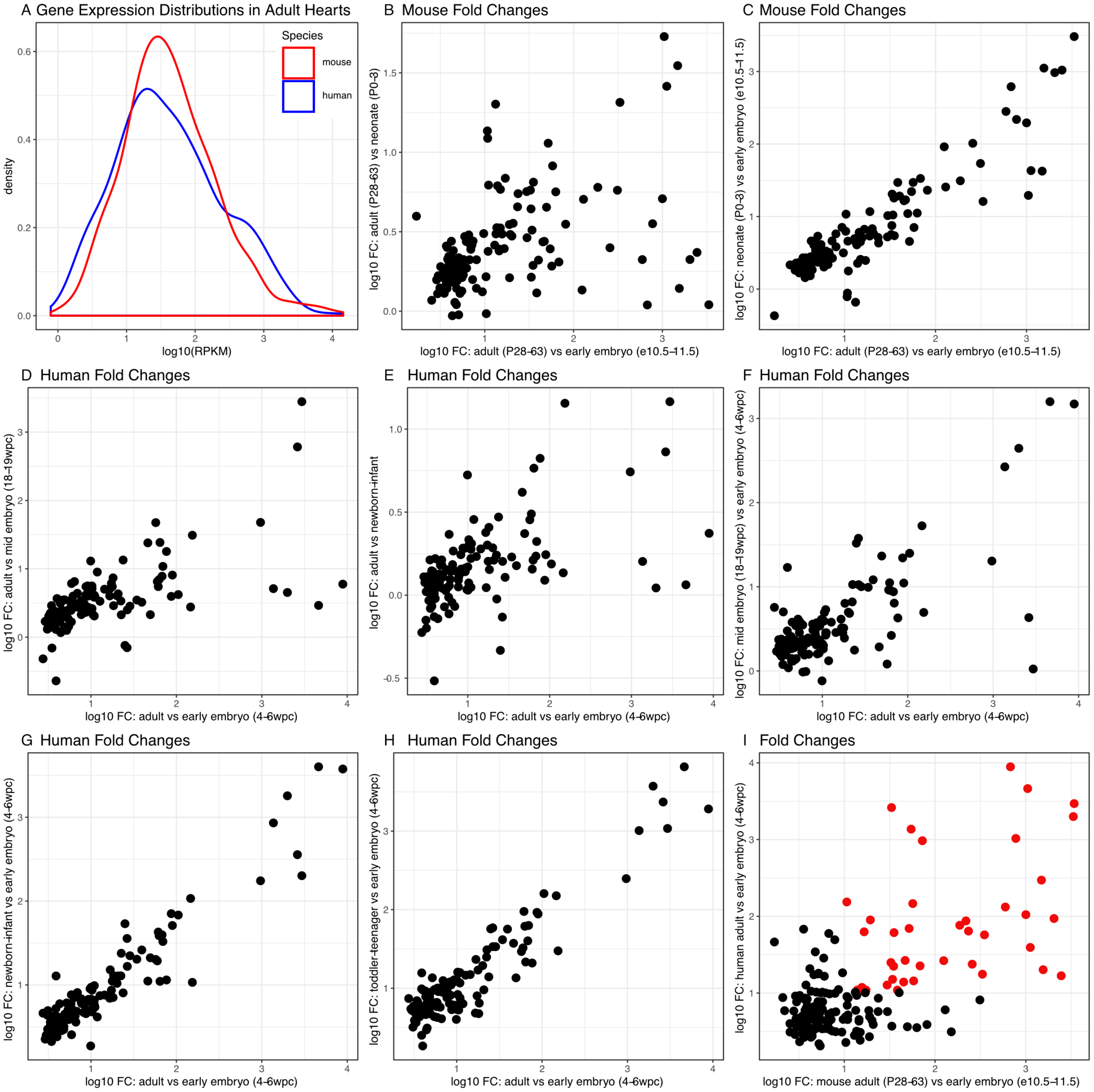

Supplementary Figure 4: Comparison of gene expression distribution and fold changes.

(A) Distribution of gene expressions shown in the base 10 logarithmic of RPKM. (B-I) Scatter plots of the base 10 logarithmic of fold changes (FCs) for (B-C) mouse hearts, (D-H) human hearts, and (I) mouse and human hearts. Comparisons are shown in plot axis. (I) Genes with at least 10-fold increases from early embryo to adult hearts in both mouse (x-axis) and human (y-axis) are shown in red. Time course plots of these genes are shown in Supplementary Figure 5 and 6.

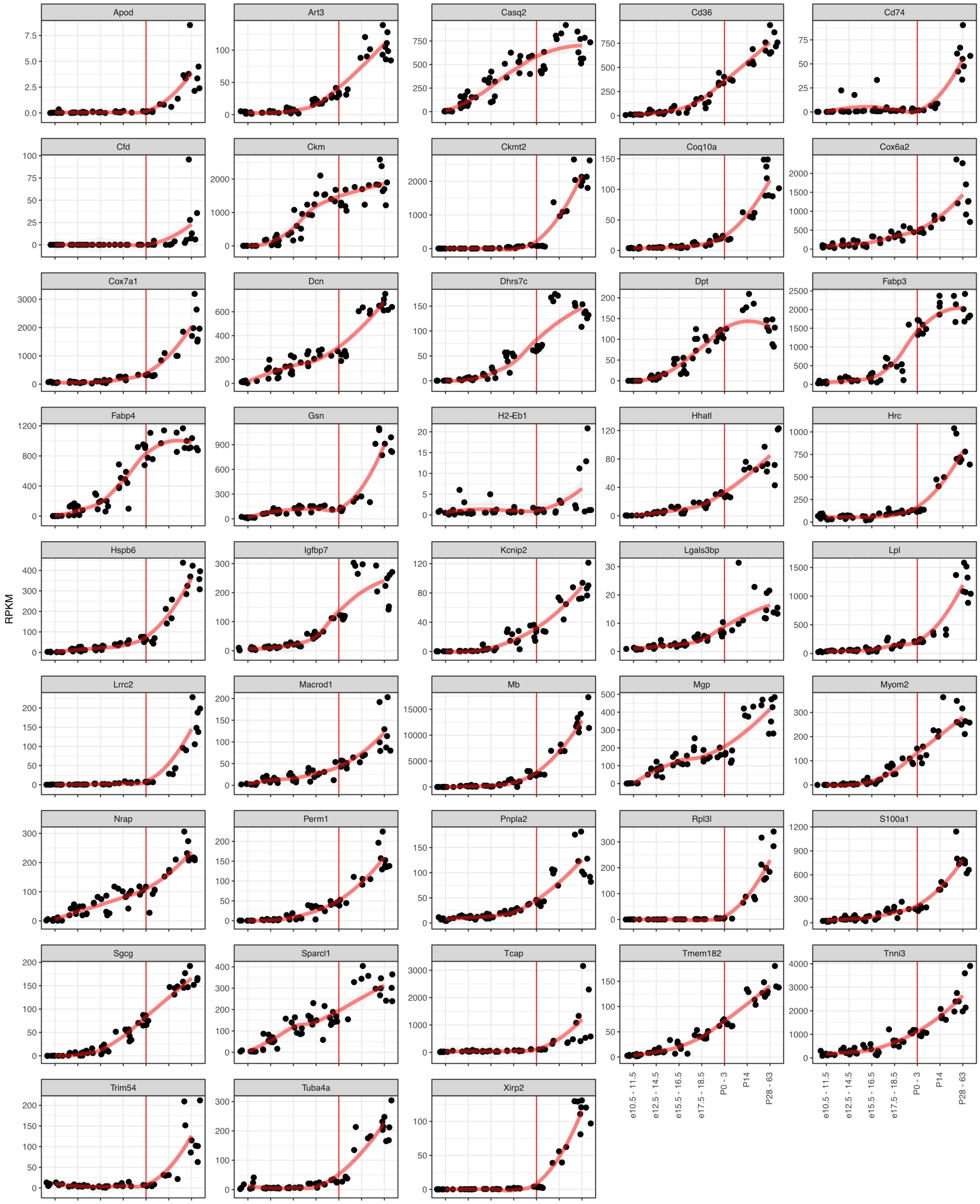

Supplementary Figure 5: Expression kinetics of candidate marker genes for cardiomyocyte maturation in mouse hearts.

Time course of gene expressions in mouse hearts are shown in RPKM. A red curve indicates local regression curve using locally estimated scatterplot smoothing (LOESS). Red vertical lines indicate the P0-3 stage.

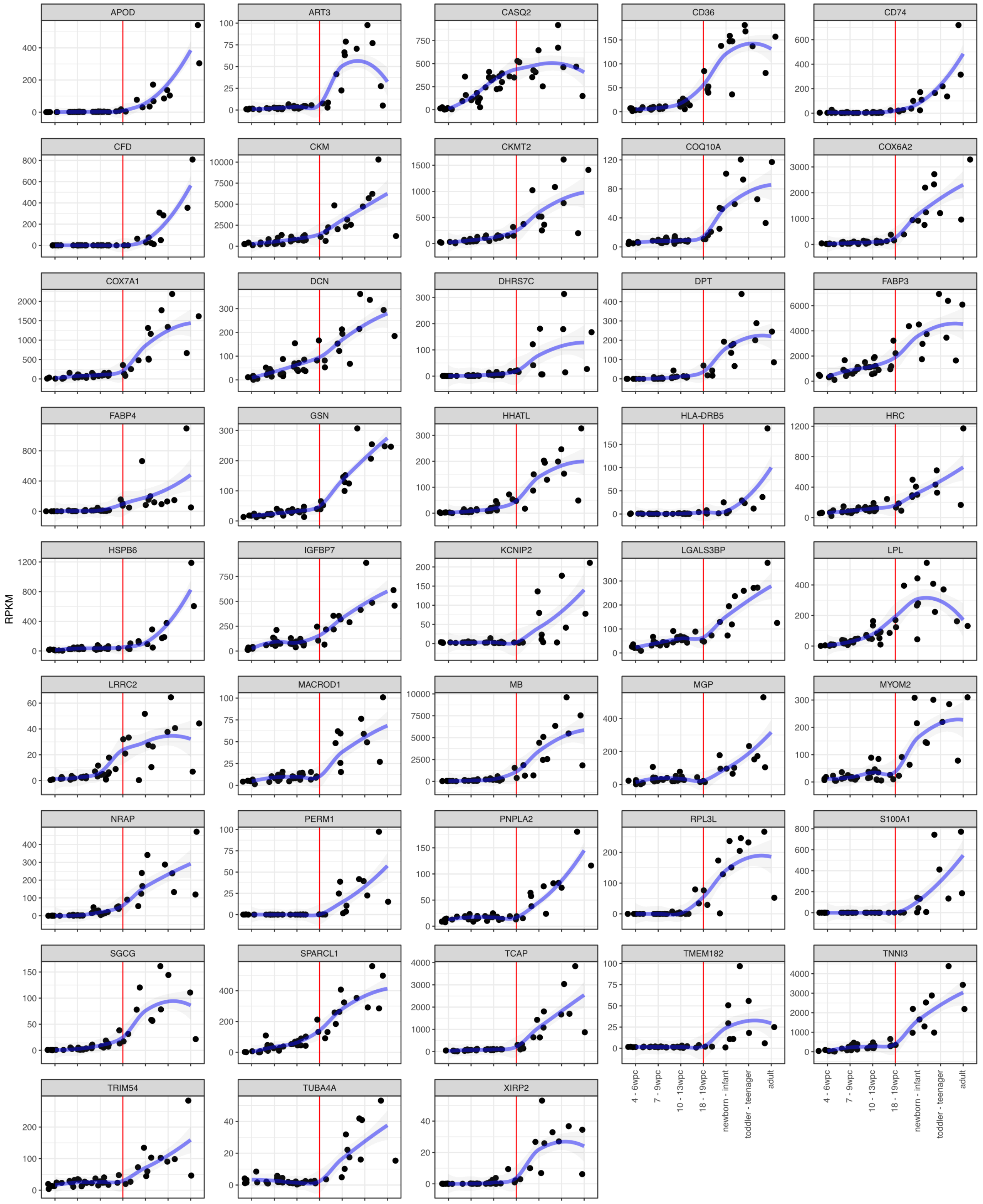

Supplementary Figure 6: Candidate marker genes for cardiomyocyte maturation in human hearts.

Time course of gene expressions in human hearts are shown in RPKM. A blue line indicates LOESS. Red vertical lines indicate the 18-19 wpc stage.
